# Association Between Out-of-Hour Admission and Short- and Long-Term Mortality in Acute Myocardial Infarction: A Systematic Review and Meta-Analysis

**DOI:** 10.3389/fcvm.2021.752675

**Published:** 2021-12-14

**Authors:** Yue-Yan Yu, Bo-Wen Zhao, Lan Ma, Xiao-Ce Dai

**Affiliations:** ^1^Department of Cardiology, Affiliated Hospital of Jiaxing University, Jiaxing, China; ^2^Department of Cardiology, First Affiliated Hospital of Zhejiang Chinese Medical University, Hangzhou, China; ^3^Department of Cardiology, Affiliated Hospital of Nantong University, Nantong, China

**Keywords:** acute myocardial infarction, on-hour, out-of-hour admission, mortality, meta-analysis, off-hour

## Abstract

**Objectives:** Out-of-hour admission (on weekends, holidays, and weekday nights) has been associated with higher mortality in patients with acute myocardial infarction (AMI). We conducted a meta-analysis to verify the association between out-of-hour admission and mortality (both short- and long-term) in AMI patients.

**Design:** This Systematic review and meta-analysis of cohort studies.

**Data Sources:** PubMed and EMBASE were searched from inception to 27 May 2021.

**Eligibility Criteria for Selected Studies:** Studies of any design examined the potential association between out-of-hour admission and mortality in AMI.

**Data Extraction and Synthesis:** In total, 2 investigators extracted the data and evaluated the risk of bias. Analysis was conducted using a random-effects model. The results are shown as odds ratios [ORs] with 95% confidence intervals (CIs). *I*^2^ value was used to estimate heterogeneity. Grading of Recommendations Assessment, Development, and Evaluation was used to assess the certainty of the evidence.

**Results:** The final analysis included 45 articles and 15,346,544 patients. Short-term mortality (defined as either in-hospital or 30-day mortality) was reported in 42 articles (15,340,220 patients). Out-of-hour admission was associated with higher short-term mortality (OR 1.04; 95%CI 1.02–1.05; *I*^2^ = 69.2%) but there was a significant statistical indication for publication bias (modified Macaskill's test *P* < 0.001). One-year mortality was reported in 10 articles (1,386,837 patients). Out-of-hour admission was also associated with significantly increased long-term mortality (OR 1.03; 95%CI 1.01–1.04; *I*^2^ = 66.6%), with no statistical indication of publication bias (*p* = 0.207). In the exploratory subgroup analysis, the intervention effect for short-term mortality was pronounced among patients in different regions (*p* = 0.04 for interaction) and socio-economic levels (*p* = 0.007 for interaction) and long-term mortality was pronounced among patients with different type of AMI (*p* = 0.0008 for interaction) or on different types of out-to-hour admission (*p* = 0.006 for interaction).

**Conclusion:** Out-of-hour admission may be associated with an increased risk of both short- and long-term mortality in AMI patients.

**Trial Registration:** PROSPERO (CRD42020182364).

## Introduction

Acute myocardial infarction (AMI) is a major cause of mortality and morbidity worldwide (1). Each year, approximately 600,000 Americans are hospitalized due to an initial episode of AMI (2). Out-of-hour admission was reported to be associated with a markedly higher mortality than admission during regular work hours in patients with AMI (3). In total, 2 meta-analyses (published in 2014 and 2016) summarized the available evidence and found that patients with AMI had a noticeably higher mortality during out of hours (4, 5). To address this issue, the UK government provided the National Health Service (NHS) hospital care as a 7-day service, which, however, did not reduce excess deaths (6). Although the association between higher mortality and out-of-hour admission appears convincing, the mechanisms remain unknown (7). Recent studies have evaluated the association of out-of-hour admission with mortality in AMI patients, but have yielded inconsistent results (8, 9). In total, 1 of these 2 studies suggested that the time effect had little impact on the high in-hospital mortality (8). The impact of ‘out-of-hours’ admission is a critical issue that could influence policy making in recruiting additional medical staff and allocating of more financial resources (10).

Although previous meta-analyses suggested that out-of-hour admission was associated with high in-hospital mortality, a recent study showed the opposite conclusion (11). Moreover, no meta-analysis has evaluated the association between out-of-hour admission and long-term mortality. Therefore, we conducted a systematic review and meta-analysis to examine whether out-of-hour admission was associated with increased mortality risk in AMI patients vs. on-hour admission.

## Methods

This meta-analysis was conducted in accordance with the Preferred Reporting Items for Systematic reviews and Meta-Analyses (PRISMA) guidelines (12). The study is registered on PROSPERO (CRD42020182364).

### Patient and Public Involvement

This is a meta-analysis based on study-level data and not an individual patient data meta-analysis. No consumer was involved in this meta-analysis.

### Search Strategy and Study Selection

We searched PubMed and EMBASE from the date of database inception to 27 May 2021. There was no language restriction in the search. Electronic search was conducted with controlled vocabulary (MeSH in PubMed and Emtree in EMBASE) and keywords as search terms. More detail is available in Supplementary Table S1. Backward snowballing (i.e., a review of references from identified articles and pertinent reviews) was used to identify additional articles.

### Eligibility Criteria

Studies were considered eligible for inclusion if they met the following criteria: (1) participants: adult patients (age≥18 years) with AMI [ST-elevation myocardial infarction (STEMI) and non-ST-elevation myocardial infarction (NSTEMI)]. Studies that included patients with unstable angina were also included. (2) intervention: out-of-hour admission; (3) outcomes: short-term (in-hospital or 30-day) mortality and long-term (1-year) mortality; and 4) types of study: randomized controlled trial or cohort study. For fully or partially duplicated publications, we retained publications with the most updated data. Out-of-hours was defined as weekends, holidays, and nights (Supplementary Tables S2A,S2B). In total, 2 investigators (Y-YY and LM) independently conducted the literature search, removed duplicate articles, read the relevant titles and abstracts, and classified records. Inconsistency was resolved through discussion.

### Data Extraction

The following data were extracted using a standardized Word (Microsoft Corporation) by 1 investigator (X-CD) and verified by another investigator (LM): author, year, country, study period, type of myocardial infarction, reperfusion therapy, outcomes, adjusted confounders, and the Newcastle-Ottawa Scale. We recorded mortality by either the number or proportion of deaths in each group and odds ratio (OR) or hazard ratio (HR) or relative risk (RR) with confidence intervals, both unadjusted and adjusted values.

### Assessment of Methodological Quality

We evaluated the methodological quality of the included cohort studies by using the Newcastle-Ottawa Scale (13). In total, 3 investigators (Y-YY, X-CD, and B-WZ) independently assessed 3 domains: (1) cohort selection, (2) comparability, and (3) outcome. The maximum score for observational studies was 9 (Supplementary Table S3). When the individuals were from communities in the same region, we upgraded the score; when the study reported important confounders, we upgraded the score; when the study reported important confounders (gender, hypertension, previous MI, smokers, diabetes mellitus, previous percutaneous coronary intervention or coronary artery bypass graft), we upgraded the score; when the study reported outcomes clearly or follow-up no less than 1 year, we upgraded the score. Cochrane risk of bias tool (RoB) (14) was used to access methodological quality of eligible RCTs or non-randomized controlled studies if included.

### Outcome Definition

The primary outcome was short-term (in-hospital or 30-day) mortality. For studies that reported both in-hospital and 30-day mortality, only in-hospital mortality was analyzed. When adjusted data were not reported, we used unadjusted data.

### Quality of Evidence

The quality of the evidence was evaluated by the Grading of Recommendations Assessment, Development, and Evaluation (GRADE) methodology, which evidence was graded as high, moderate, low, or very low. In total, 2 investigators independently performed GRADE assessments for every outcome. RCT was initially considered as grade of high or observational study was initially consider as grade of low and was downgraded based on the following criteria: risk of bias, inconsistency (substantial unexplained heterogeneity, I^2^ > 50%, and *P* < 0.10), indirectness (presence of factors that limit the generalizability of the results), imprecision (the 95% confidence interval for effect estimates overlap the minimally important differences for benefit or harm), and publication bias (significant evidence of a small study effect) or upgraded. The GRADE Profiler (Windows-only tool, GRADEpro) was used to construct summary tables.

### Statistical Analysis

Pooled ORs and their corresponding 95% confidence intervals (CIs) for categorical variables (dichotomous outcomes) were calculated using a random-effects model because of the effect of clinical and methodological heterogeneity among studies, with the inverse variance method. We calculated adjusted or unadjusted ORs with 95% CIs for the overall effect estimate. HR was considered equivalent to OR. RR was converted to OR using the following equation: (OR = RR × (1-pRef)/[1-RR × pRef]), where pRef is the prevalence of the outcome in the reference group (15). *I*^2^ value was used to estimate heterogeneity. *I*^2^ < 25%, 50%, or >50% indicated mild, moderate, or substantial heterogeneity, respectively (16). Publication bias was assessed by visual inspection of a contour-enhanced funnel plot (17), and examined by a modified Macaskill test (18). Subgroup analyses were conducted based on data adjustment (adjusted vs. unadjusted), type of AMI (STEMI vs. NSTEMI), type of out-of-hours (weekends, holidays, and night vs. weekends and holidays vs. night), region (North America vs. Europe vs. Asia and others), socio-economic level of countries (developing countries vs. developed countries) and years of publication based on different quality of care and prognosis setting every 10 years (2001–2010 vs. 2011–2020). We also conducted sensitivity analyses where data that had been adjusted for important confounders were compared to unadjusted data. Cumulative meta-analysis was used to evaluated whether the result was robust. Statistical significance was set at *P* < 0.05 (2-sided). All statistical analyses were conducted in STATA (Stata Version 16.0; Stata Corp) and Review Manager (Version 5.3; Cochrane Collaboration).

## Results

### Baseline Features of Eligible Studies

**Figure 1:**
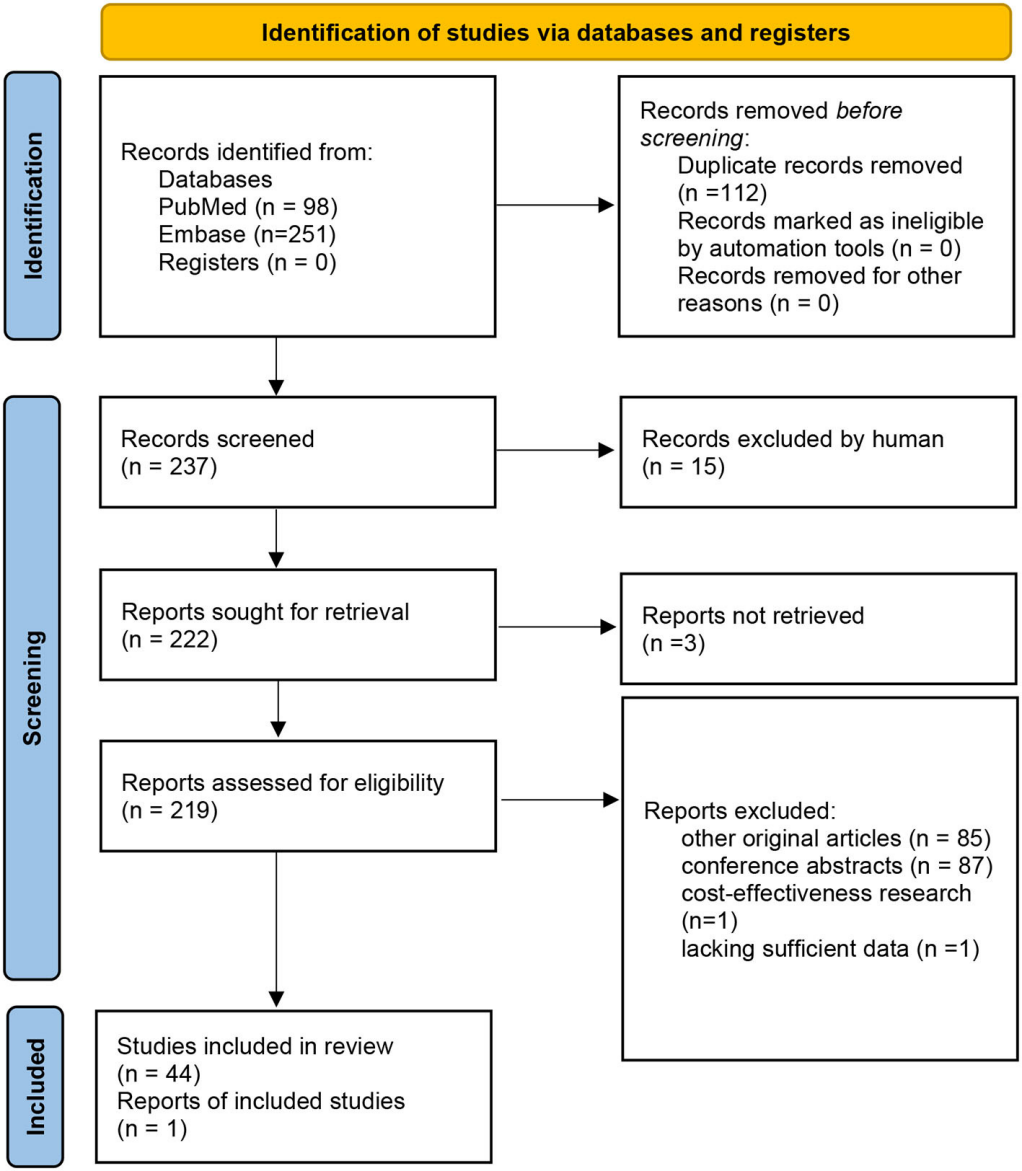
Preferred reporting items for systematic reviews and meta-analyses flow diagram.

The meta-analysis included 45 articles (all cohort studies), with a total of 15,346,544 patients (3, 8, 9, 11, 19–59) (Figure 1). The characteristics of the included studies are summarized in Supplementary File 2. In total, 22 articles (1,507,570 patients) reported data on STEMI; 7 articles (4,034,490 patients) reported data on NSTEMI; and 16 articles (1,935,531 patients) did not separate STEMI and NSTEMI. Short-term mortality was reported by 42 articles (15,339,920 patients). One-year mortality was reported by 10 articles (1,386,837 patients). For 1-year mortality, five studies with 34,004 patients reported data on STEMI, 2 studies with 45,885 patients reported results on NSTEMI, and 4 studies totaling 1,306,948 patients did not report results on STEMI and NSTEMI separately.

### Risk of Bias and Funnel Plot

The risk of bias is low to moderated. The bias score was 7 in 20 studies, 8 in 22 studies, and 9 in the remaining 3 studies (Supplementary Table S2). Contour-enhanced funnel plots showed asymmetry for short- (modified Macaskill test, *P* < 0.001) but not for long-term (modified Macaskill test, P = 0.207) mortality (Supplementary Figures S1A,B).

### Short-Term Mortality

**Figure 2:**
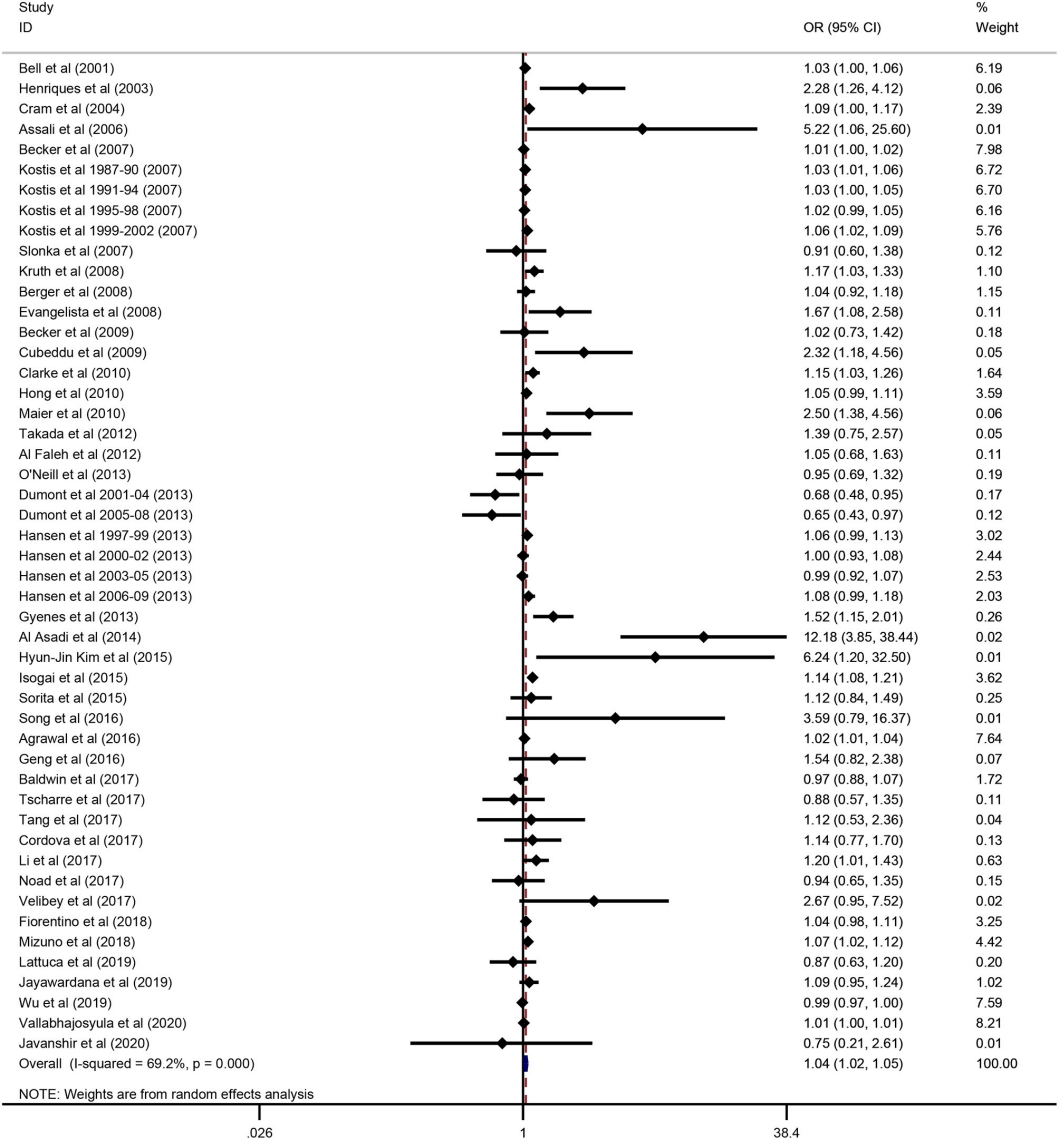
Odds Ratio for short-term AMI mortality during out-of-hours vs. on-hour.

Thirty studies reported in-hospital mortality and 10 studies reported 30-day mortality. In the overall analysis, compared with on-hour admission, out-of-hour admission was associated with a significantly higher short-term mortality (OR 1.04; 95% CI 1.02–1.05; *P* < 0.001; *I*^2^ = 69.2%; Figure 2).

We only performed an exploratory subgroup analysis because of unavoidable ecological bias. The exploratory subgroup analysis revealed that higher short-term mortality was associated with regions (North America in 11 studies: OR 1.02; 95% CI 1.01–1.03; *P* < 0.001; *I*^2^ = 55%; Europe in 13 studies: OR 1.03; 95% CI 0.98–1.08; *P* = 0.20; *I*^2^ = 62%; Asia and others in 18 studies: OR 1.13; 95% CI 1.05–1.22; *P* = 0.002; *I*^2^ = 65%; *P* = 0.04 for interaction). Social-economic level of countries was associated with short-term mortality (developed countries in 39 studies: OR 1.04; 95% CI 1.02–1.05; developing countries in 10 studies: OR 1.45; 95% CI 1.13–1.84; *P* = 0.007 for interaction). The other subgroup results showed no statistical significance (All *P* > 0.05 for interaction) (Table 1).

**Table 1:** Subgroup analysis of short-term mortality of acute myocardial infarction patients during out-of-hour admission vs. one-hour admission.

**Subgroup**	**No. of studies**	**Odds ratio (95%CI)**	***I^**2**^* (%)**	***P* value**	***P* value for interaction**
All studies	49	1.04 (1.02–1.05)	69.2%	<0.001	
**Adjustment:**					
Adjusted data	23	1.04 (1.02–1.06)	70%	<0.001	0.90
Unadjusted data	26	1.04 (1.00–1.08)	63%	0.02	
**Diagnosis:**					
STEMI	24	1.08 (1–1.16)	76%	0.04	0.16
NSTEMI	10	1.02 (0.98–1.06)	66%	0.36	
**out-of-hour time frame:**					
Weekends, holidays, and nights	19	1.11 (0.98–1.27)	68%	0.11	0.19
Weekends and holidays	26	1.03 (1.02–1.05)	61%	<0.001	
Nights	4	1.58 (0.90–2.78)	77%	0.11	
**Region:**					
North America	14	1.02 (1.01–1.03)	55%	<0.001	0.04
Europe	17	1.03 (0.98–1.08)	62%	0.20	
Asia and others	18	1.13 (1.05–1.22)	65%	0.002	
**Socio-economic level of countries:**					
Developed countries	39	1.04 (1.02–1.05)	71%	<0.001	0.007
Developing countries	10	1.45 (1.13–1.84)	72%	0.003	
**Years of publication:**					
2001–2010	18	1.05 (1.02–1.07)	71%	<0.001	0.48
2011–2020	31	1.04 (1.01–1.06)	70%	0.005	

### Long-Term Mortality

**Figure 3:**
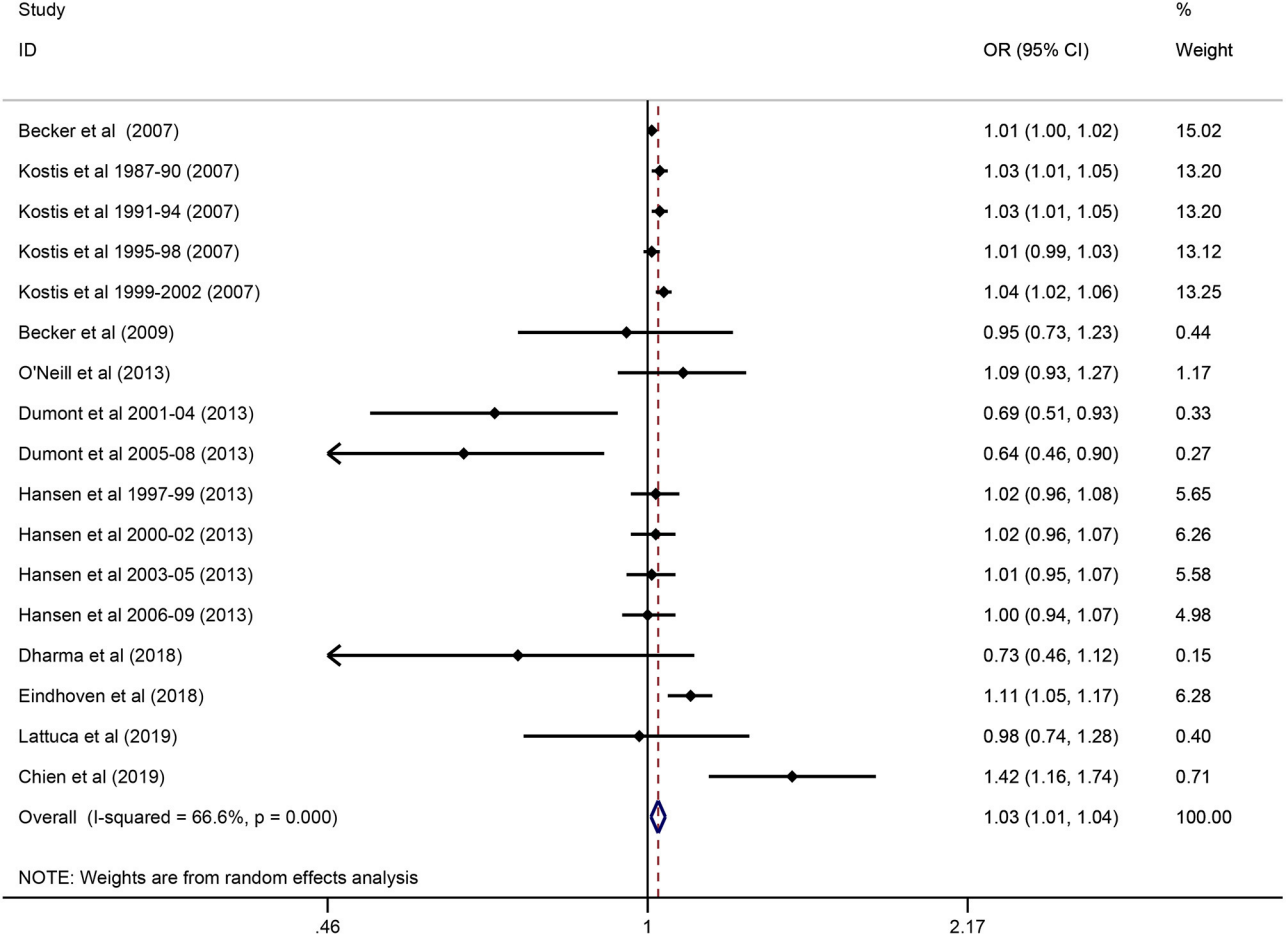
Odds Ratio for long-term AMI mortality during out-of-hours vs. on-hour.

In total, 10 studies reported 1-year mortality. In the overall analysis, compared with 1-hour admission, out-of-hour admission was associated with a significantly higher 1-year mortality (OR 1.03; 95% CI 1.01–1.04; *P* = 0.004; *I*^2^ = 66.6%; Figure 3).

Exploratory subgroup analysis revealed a more pronounced intervention effect among patients with the type of AMI (NSTEMI in 2 studies: OR 1.18; 95% CI 1.07–1.29; *P* = 0.001; *I*^2^ = 28.8%; STEMI in 5 studies: OR 0.85; 95% CI 0.73–1.01; *P* = 0.058; *I*^2^ = 59.9%; *P* = 0.0008 for interaction) (Table 2). Types of out-of-hour admission showed an association with mortality (*p* = 0.006 for interaction): (1) weekends and holidays and night were associated with a reduction in the risk of mortality (OR 0.81; 95% CI 0.67–0.96; *P* = 0.017; *I*^2^ = 38.2%;4 studies); (2) weekends and holidays was associated with a higher mortality (OR 1.03; 95% CI 1.01–1.04; *P* < 0.000; *I*^2^ = 67.4%;5 studies) (Table 2). No significant difference in the intervention effect was observed according to the other subgroups (all *P* > 0.05 for interaction) (Table 2).

**Table 2:** Subgroup analysis of long-term mortality due to acute myocardial infarction patients during off-hour admission vs. one-hour admission.

**Subgroup**	**No. of studies**	**Odds ratio (95%CI)**	***I^**2**^* (%)**	***P* value**	***P* value for interaction**
All studies	17	1.03 (1.01–1.04)	66.6%	0.004	
**Adjustment:**					
Adjusted data	10	1.03 (1.02–1.04)	0%	<0.001	0.72
Unadjusted data	17	1.01 (0.91–1.12)	83.2%	0.875	
**Diagnosis:**					
STEMI	5	0.85 (0.73–1.01)	59.9%	0.058	0.001
NSTEMI	2	1.18 (1.07–1.29)	28.8%	<0.001	
**Out-of-hour time frame:**					
Weekends, holidays, and nights	12	0.81 (0.67–0.96)	38.2%	0.017	0.006
Weekends and holidays	5	1.03 (1.01–1.04)	67.4%	<0.001	
**Region:**					
North America	6	1.02 (1.01–1.04)	55.5%	<0.001	0.99
Europe	9	1.00 (0.95–1.06)	66.4%	0.855	
Asia and others	2	1.05 (0.55–2.01)	85.9%	0.883	
**Socio-economic level of countries:**					
Developed countries	15	1.02(1.01–1.04)	87%	0.007	
Developing countries	2	1.05 (0.55–2)	61%	0.89	0.95
**Years of publication:**					
2001–2010	6	1.02 (1.01–1.03)	55%	<0.001	
2011–2020	11	1.02 (0.96–1.08)	72%	0.46	0.98

### Sensitivity Analysis

By adjusting for important confounders, the sensitivity analyses (Supplementary Table S4) confirmed that the association between short-term mortality and AMI did not change with the use of random-effects models for the meta-analysis. In cumulative meta-analysis, we found that out-of-hour admission was associated with a slightly higher (1–3%) odds of short-term mortality (Supplementary Figure S2), which meant the result was robust.

## Discussion

### Main Findings

The present meta-analysis of out-of-hour admission studies for AMI substantially expand the results of the former 2 meta-analyses (4, 5) which reports that out-of-hour admission is associated with higher one-year mortality. In total, 3 principal findings are summarized: For in-hospital or 30-day period, out-of-hour admission is associated with an increased risk of mortality in AMI patients, which is affected by regions and socio-economic level of countries; for 1-year follow-up, NSTEMI patients have high mortality in out-of-admission but not for STEMI patients; for 1-year follow-up, weekends and holidays increased the odds of mortality in AMI patients but weekends and holidays and night showed a reduction in the odds of the mortality in AMI patients. Although the association between mortality and out-of-hour admission was statistically significant, these findings should be used cautiously. We suggested that the large sample had a major impact on the results because countries from different regions had different socio-economic levels which meant the treatment ability of those hospitals were different. Based on GRADE evidence assessment, the strength of evidence was very low for short- and long-term mortality in AMI (Supplementary Table S5).

### Possible Mechanisms for Findings

Out-of-hour admission is a time-effect factor, and many other confounders have an impact on it. The exploratory subgroup analyses in our meta-analysis showed false-positive results (different out-of-hour types in short-term mortality) or false-negative results (different out-of-hour types in long-term mortality) which meant an existing ecological bias, and this might be influenced by the substantial heterogeneity, the number of included studies, and the very low evidence strength. Ecological bias cannot be avoided unless data analysis is based on individual-level patient data. Several studies (39, 41) suggest that hospitals with less experienced doctors and staff or differences in accessibility lead to higher mortality during out-of-hour admission than on-hours admissions. It has been shown that adequate staffing of nurses may reduce the mortality of patients (46). Out-of-hour admission may at most only partially explain the increased mortality of AMI patients because the mortality does not decrease even with improved clinical standards (10). The door-to-balloon time may not be a key factor in out-of-hour admission (55) because our study showed that short-term mortality for patients with STEMI during out-of-hour and on-hour admission has no statistically significantly different in the subgroup analysis. Recently, high-volume hospitals have been ensuring that treatment conforms to guidelines, so the short-term mortality of STEMI should be no different from on-hour admission mortality (55). Nevertheless, our findings showed that the one-year mortality for patients with NSTEMI was higher than that for patients with STEMI, which might be caused by a lower rate of primary percutaneous coronary intervention. Walker et al. (7) reported for the first time that patient-level factors such as biochemical and hematological parameters were associated with higher mortality. However, out-of-hour admission may still partially contribute to increased mortality (60).

AMI, especially STEMI, is a time-sensitive illness, and a shorter time to treat these patients can increase their survival. Most of these patients have the first episode of AMI, and they may not quickly present to the emergency department because of the lack of recognition of this disease. This may explain why elderly patients (over 65 years old) have a higher mortality than younger patients (56). Several lifestyle-related factors (61), including physical activity, alcohol use, and emotional stress, may also influence mortality. To fully tackle the higher out-of-hour admission mortality faces challenges because potential factors are unknown.

### Implications for Clinical Practice

Our findings have important implications for clinicians and policy makers because AMI is so common that we need to have the right approach to tackle this disease. Our meta-analysis found that the risk of both short- and long-term mortality was approximately 3% higher during out-of-hour admission than during one-hour admission. However, it may be an overestimated result because our results show that the mortality of developing countries significantly higher than the counterpart in developed countries. This may be the reason which causes the overall increase in mortality. An appropriate management process is needed for AMI patients from the community to the hospital. To improve public awareness campaigns, community physicians or health care providers should provide locals, especially those who are elderly, with brochures or access to health salons. In developing countries, hospitals should ensure the following: (1) the cath-lab can be activated in a timely manner; (2) experienced doctors and nurses and other medical staff are sufficient during off time. In North America and Europe, hospital-level factors do not seem to be the main reason for the increased mortality (10). It may be more important to identify unknown factors and then devise evidence-based policies.

### Limitations

This meta-analysis has some major limitations. First, the substantial heterogeneity associated with pooled estimates for short- and long-term mortality may have reduced the quality of evidence. Second, we found substantial heterogeneity in subgroup analyses, which, however, cannot explain all these results because most of them showed no statistical significance. We think that the number of studies and substantial heterogeneity may influence the results. Third, the definition of out-of-hour admission varied country by country; hence, the results may not be applicable to all included countries. Fourth, several studies reported only AMI and did not include specific results regarding whether STEMI was associated with a higher mortality. Fifth, we tried to find a method to evaluate the severity of illness among the included studies, but no reference reported an appropriate method to define how many individuals in the cohort can be reviewed as critically ill. Therefore, we did not stratify patients by severity of illness. Sixth, we did not carry out subgroup analysis of patients according to gender and age, which may have impacted the quality of evidence. Seventh, most of the included studies focused on PCI, and a small number of studies included different treatment approaches, such as fibrinolysis. Eighth, for 1-year mortality, only 2 studies report the NSTEMI, and more studies are needed to reevaluate the result. So, we cannot explore the precise results concerning the relationship between the out-of-hour admission and treatment approaches in STEMI and NSTEMI.

## Conclusion

In conclusion, out-of-hour admission may be associated with higher short-or long-term mortality in AMI. Out-of-hour admission is associated with higher short-term mortality only in some countries.

## Data Availability Statement

The original contributions presented in the study are included in the article/Supplementary Material, further inquiries can be directed to the corresponding authors.

## Author Contributions

X-CD: analysis and interpretation of data, drafting the article, reviewing, and editing the article. Y-YY and X-CD: conception and design of the study, acquisition of data, making the GRADE assessment (Y-YY and X-CD received training), and final approval of the version to be published. LM and B-WZ: drafting the manuscript, checking the methodology, and curating the data. All authors contributed to the article and approved the submitted version.

## Funding

This work was supported by Provincial-Municipal Joint Construction of Key Medical Disciplines in Zhejiang Province (2019-ss-xxgbx) and Jiaxing Key Innovation Team Fund (2018-xjqxcxtd).

## Conflict of Interest

The authors declare that the research was conducted in the absence of any commercial or financial relationships that could be construed as a potential conflict of interest.

## Publisher's Note

All claims expressed in this article are solely those of the authors and do not necessarily represent those of their affiliated organizations, or those of the publisher, the editors and the reviewers. Any product that may be evaluated in this article, or claim that may be made by its manufacturer, is not guaranteed or endorsed by the publisher.

## Abbreviations

AMI: acute myocardial infarction
NHS: National Health Service
STEMI: ST-segment elevation myocardial infarction
NSTEMI: non-ST-segment elevation myocardial infarction
RISMA: Preferred Reporting Items for Systematic reviews and Meta-Analyses
OR: odds ratio
CIs: confidence interval.
