# Supplementary material for: Association Between Out-of-Hour Admission and Short- and Long-Term Mortality in Acute Myocardial Infarction: A Systematic Review and Meta-Analysis
Source: Front Cardiovasc Med. 2021 Dec 14;8:752675. doi: 10.3389/fcvm.2021.752675 (PMC8712470; doi:10.3389/fcvm.2021.752675)
Supplement: Supplementary file 1 [file Data_Sheet_1.PDF]

Appendix Tables and Figures

Appendix Table S1. Details of Search Strategy

Appendix Figure S1A. Contour-enhanced Funnel Plot for Short-Term Mortality

Appendix Figure S1B. Contour-enhanced Funnel Plot for Long-Term Mortality

Appendix Figure S2. Cumulative Meta-analysis for Short-Term Mortality

Appendix Table S2A. Type of Off-hours

Appendix Table S2B. Details of Included Studies about Type of Off-hours

Appendix Table S3. Quality Assessment of the Eligible Cohort Studies

Appendix Table S4. Sensitive Analysis for short-Term Mortality

Appendix Table S5. GRADE evidence profile

Appendix Table S1. Details of Search Strategy
Source: PubMed; search on: May 27, 2021; Results: 98

Search	Query	Items
#1	“Myocardial Infarction” [Mesh]	173,143
#2	“Myocardial Infarction*” [Title/Abstract]	180,582
#3	“Myocardial Infarct*”[Title/Abstract]	195,092
#4	“NSTEMI” [Title/Abstract]	2,326
#5	“STEMI”[Title/Abstract]	10,573
#6	“non-ST-segment elevation myocardial infarction” [Title/Abstract]	1,507
#7	“ST-segment elevation myocardial infarction” [Title/Abstract]	8,015
**#8**	**#1 OR #2 OR #3 OR #4 OR #5 OR #6 OR #7**	**249,379**
#9	“Night” [Title/Abstract]	63,791
#10	“Nighttime” [Title/Abstract]	9,514
#11	“weekends” [Title/Abstract]	4,204
#12	“off-hours” [Title/Abstract]	352
#13	“off-hour” [Title/Abstract]	98
#14	“out-of-hours” [Title/Abstract]	1,788
#15	“out-of-hour” [Title/Abstract]	35
#16	“after-hours” [Title/Abstract]	1,332
#17	“after-hour” [Title/Abstract]	108
**#18**	**#9 OR #10 OR #11 OR #12 OR #13 OR #14 OR #15 OR #16 OR #17**	**77,211**
#19	“admission*” [Title/Abstract]	211,657
#20	“admit*” [Title/Abstract]	209,106
**#21**	**#19 OR #20**	**365,507**
#22	“Mortality” [Title/Abstract]	741,696
#23	“death*” [Title/Abstract]	808,636
#24	“Survival” [Title/Abstract]	919,703
**#25**	**#22 OR #23 OR #24**	**2,074,806**
**#26**	**#8 AND #18 AND #21AND #25**	**95**
**#27**	**From 21/03/2020-27/05/2021**	**3**
**#28**	**#26 OR #27**	**98**

Source: EMBASE; search on: May 27, 2021; Results: 251

Search	Query	Items
#1	“heart infarction” [exp/mj]	177,441
#2	“Myocardial Infarction*” [ti,ab,kw]	282.342
#3	“Myocardial Infarct*” [ti,ab,kw]	294,855
#4	“non st segment elevation myocardial infarction” [ti,ab,kw]	2,209
#5	“NSTEMI” [ti,ab,kw]	7,281
#6	“st-segment elevation myocardial infarction” [ti,ab,kw]	12,900
#7	“STEMI” [ti,ab,kw]	27,478
**#8**	**#1 OR #2 OR #3 OR #4 OR #5 OR #6 OR #7**	**335,242**
#9	“Night” [ti,ab,kw]	100,816
#10	“Nighttime” [ti,ab,kw]	14,479
#11	“weekends” [ti,ab,kw]	6,865
#12	“off-hours” [ti,ab,kw]	630
#13	“off-hour” [ti,ab,kw]	206
#14	“out of hours” [ti,ab,kw]	3247
#15	“out of hour” [ti,ab,kw]	87
#16	“after hours” [ti,ab,kw]	1,936
#17	“after hour” [ti,ab,kw]	190
**#18**	**#9 OR #10 OR #11 OR #12 OR #13 OR #14 OR #15 OR #16 OR #17**	**121,700**
#19	“admission*” [ti,ab,kw]	375,278
#20	“admit*” [ti,ab,kw]	375,496
**#21**	**#19 OR #20**	**641,711**
#22	“Mortality” [ti,ab,kw]	1,122,726
#23	“death*” [ti,ab,kw]	1,198,768
#24	“Survival” [ti,ab,kw]	1,388,227
**#25**	**#22 OR #23 OR #24**	**3,073,850**
**#26**	**#8 AND #18 AND #21 AND #25**	**225**
**#27**	**From 27/03/2020-27/05/2021**	**26**
**#28**	**#26 OR #27**	**251**

Appendix Table S2A. Type of Off-hours

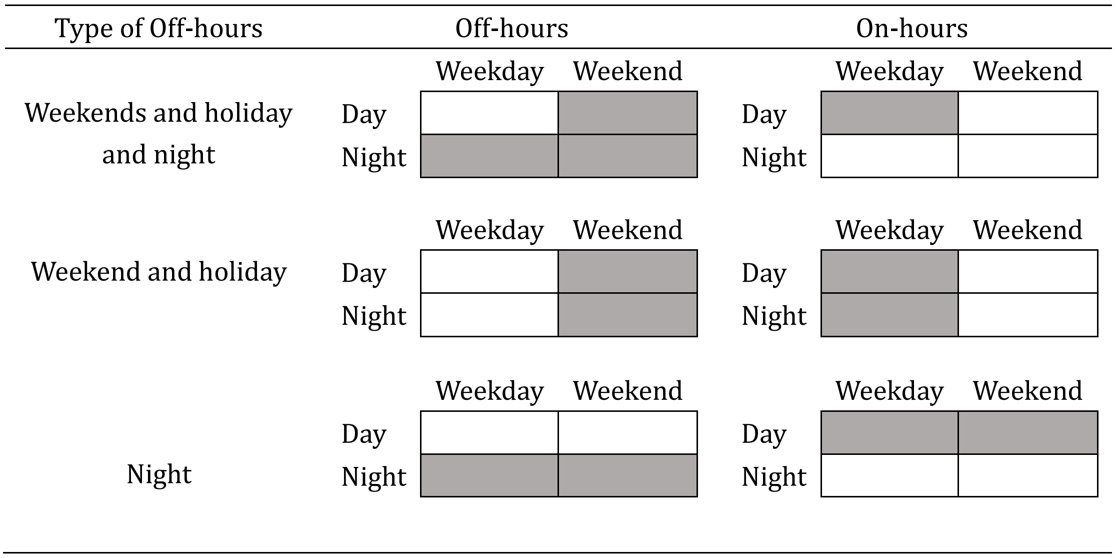

Off-hours: Weekends and holiday and night as OFF-1
Weekend and holiday as OFF-2
Night as OFF-3

On-hours: weekday’s day as ON-1
weekdays’ day and night as ON-2
weekday and weekend’s day as ON-3

Appendix Table S2B. Details of Included Studies about Type of Off-hours

Author and Year	Off-hours	On-hours
Kruth et al 2008	OFF-1	ON-1
Lattuca et al 2019	OFF-1	ON-1
O'Neill et al 2013	OFF-2	ON-1
Song et al 2016	OFF-1	ON-1
Tang et al 2017	OFF-2	ON-2
Agrawal et al 2016	OFF-2	ON-2
Bell et al 2001	OFF-2	ON-2
Berger et al 2008	OFF-1	ON-1
Baldwin et al 2017	OFF-2;OFF-3	ON-2;ON-3
Jayawardana et al 2019	OFF-1	ON-1
Cordova et al 2017	OFF-2	ON-2
Dharma et al 2018	OFF-1	ON-1
Dumont et al 2013	OFF-1	ON-1
Eindhoven et al 2018	OFF-2	ON-2
Takada et al 2012	OFF-3	ON-3
Fiorentino et al 2018	OFF-2	ON-2
Geng et al 2016	OFF-1	ON-1

Gyenes et al 2013	OFF-2	ON-2
Hansen et al 2013	OFF-2	ON-2
Henriques et al 2003	OFF-3	ON-3
Becker et al 2007	OFF-2	ON-2
Isogai et al 2015	OFF-2	ON-2
Hyun-Jin Kim et al 2015	OFF-2	ON-2
Kostis et al 2007	OFF-2	ON-2
Li et al 2017	OFF-1	ON-1
Maier et al 2010	OFF-1	ON-1
Chien et al 2019	OFF-2	ON-2
Al-Asadi et al 2014	OFF-1	ON-1
Noad et al 2017	OFF-2	ON-2
Wu et al 2019	OFF-3	ON-3
Mizuno et al 2018	OFF-2;OFF-3	ON-2;ON-3
Sorita et al 2015	OFF-1	ON-1
Tscharre et al 2017	OFF-1	ON-1
Velibey et al 2017	OFF-1	ON-1
Cubeddu et al 2009	OFF-1	ON-1
Al Faleh et al 2012	OFF-1	ON-1
Assali et al 2006	OFF-3	ON-3
Becker et al 2009	OFF-1	ON-1
Clarke et al 2010	OFF-2	ON-2
Cram et al 2004	OFF-2	ON-2
Evangelista et al 2008	OFF-2	ON-2
Hong et al 2010	OFF-2	ON-2
Slonka et al 2007	OFF-1	ON-1
Vallabhajosyula et al 2020	OFF-2	ON-2
Javanshir et al 2020	OFF-1	ON-1

Appendix Table S3. Quality Assessment of the Eligible Cohort Studies

	Cohort Selection			Comparability			Outcome		
Author	Exposed Group represents average in community	Nonexposed represents the same community as exposed	Ascertain exposure through records or structured interviews	Demonstration that outcomes not present at start of study	Adjusted by prehospital delay	Adjusted by any other factors	Confirm outcome via secure record	Followup long enough for outcome to occur	Loss to followup <10%
Kruth et al, 2008	✓	✓	✓	✓	✓	✓	✓	✓	✓
Lattuca et al, 2019	✓	✓	✓	✓			✓	✓	✓
O'Neill et al, 2013	✓	✓	✓	✓		✓	✓	✓	✓
Song et al, 2016	✓	✓	✓	✓			✓	✓	✓
Tang et al, 2017	✓	✓	✓	✓			✓	✓	✓
Agrawal et al,2016	✓	✓	✓	✓		✓	✓	✓	✓
Bell et al, 2001	✓	✓	✓	✓		✓	✓	✓	✓
Berger et al, 2008	✓	✓	✓	✓	✓	✓	✓	✓	✓
Baldwin et al, 2017	✓	✓	✓	✓		✓	✓	✓	✓
Jayawardana et al, 2019	✓	✓	✓	✓		✓	✓	✓	✓
Cordova et al, 2017	✓	✓	✓	✓			✓	✓	✓
Dharma et al, 2018	✓	✓	✓	✓		✓	✓	✓	✓
Dumont et al, 2013	✓	✓	✓	✓			✓	✓	✓
Eindhoven et al, 2018	✓	✓	✓	✓			✓	✓	✓
Takada et al, 2012	✓	✓	✓	✓			✓	✓	✓
Fiorentino et al, 2018	✓	✓	✓	✓			✓	✓	✓
Geng et al, 2016	✓	✓	✓	✓			✓	✓	✓

Gyenes et al, 2013	✓	✓	✓	✓		✓	✓	✓	✓
Hansen et al, 2013	✓	✓	✓	✓		✓	✓	✓	✓
Henriques et al, 2003	✓	✓	✓	✓		✓	✓	✓	✓
Becker et al, 2007	✓	✓	✓	✓			✓	✓	✓
Isogai et al, 2015	✓	✓	✓	✓		✓	✓	✓	✓
Hyun-Jin Kim et al, 2015	✓	✓	✓	✓			✓	✓	✓
Kostis et al, 2007	✓	✓	✓	✓		✓	✓	✓	✓
Li et al, 2017	✓	✓	✓	✓			✓	✓	✓
Maier et al, 2010	✓	✓	✓	✓		✓	✓	✓	✓
Chien et al, 2019	✓	✓	✓	✓			✓	✓	✓
Al-Asadi et al, 2014	✓	✓	✓	✓		✓	✓	✓	✓
Noad et al, 2017	✓	✓	✓	✓			✓	✓	✓
Wu et al, 2019	✓	✓	✓	✓		✓	✓	✓	✓
Mizuno et al, 2018	✓	✓	✓	✓			✓	✓	✓
Sorita et al, 2015	✓	✓	✓	✓		✓	✓	✓	✓
Tscharre et al, 2017	✓	✓	✓	✓		✓	✓	✓	✓
Velibey et al, 2017	✓	✓	✓	✓			✓	✓	✓
Cubeddu et al, 2009	✓	✓	✓	✓		✓	✓	✓	✓
Al Faleh et al, 2012	✓	✓	✓	✓			✓	✓	✓
Assali et al, 2006	✓	✓	✓	✓	✓		✓	✓	✓
Becker et al, 2009	✓	✓	✓	✓		✓	✓	✓	✓

Clarke et al,2010	✓	✓	✓	✓		✓	✓	✓	✓
Cram et al, 2004	✓	✓	✓	✓			✓	✓	✓
Evangelista et al, 2008	✓	✓	✓	✓			✓	✓	✓
Hong et al, 2010	✓	✓	✓	✓		✓	✓	✓	✓
Slonka et al, 2007	✓	✓	✓	✓			✓	✓	✓
Vallabhajosyula et al 2020	✓	✓	✓	✓	✓		✓	✓	✓
Javanshir et al 2020	✓	✓	✓	✓	✓	✓	✓	✓	✓

Appendix Figure S1A. Contour-enhanced Funnel Plot for Short-Term Mortality

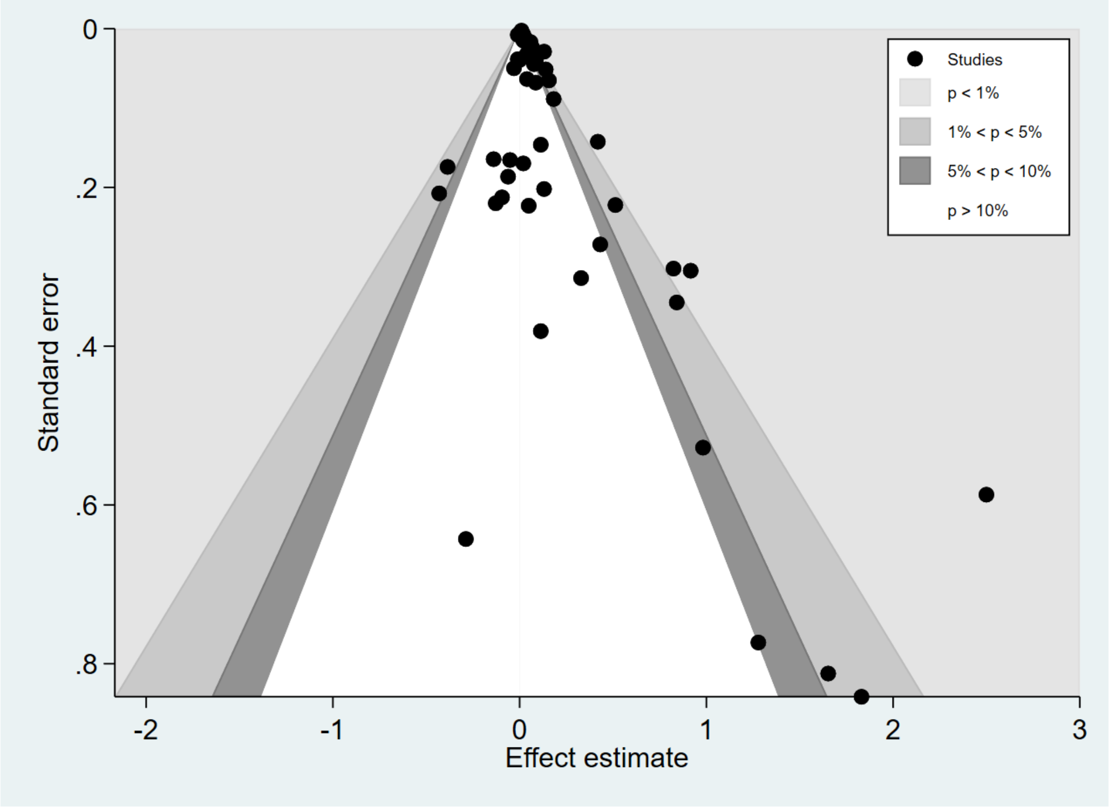

Appendix Figure S1B. Contour-enhanced Funnel Plot for Long-Term Mortality

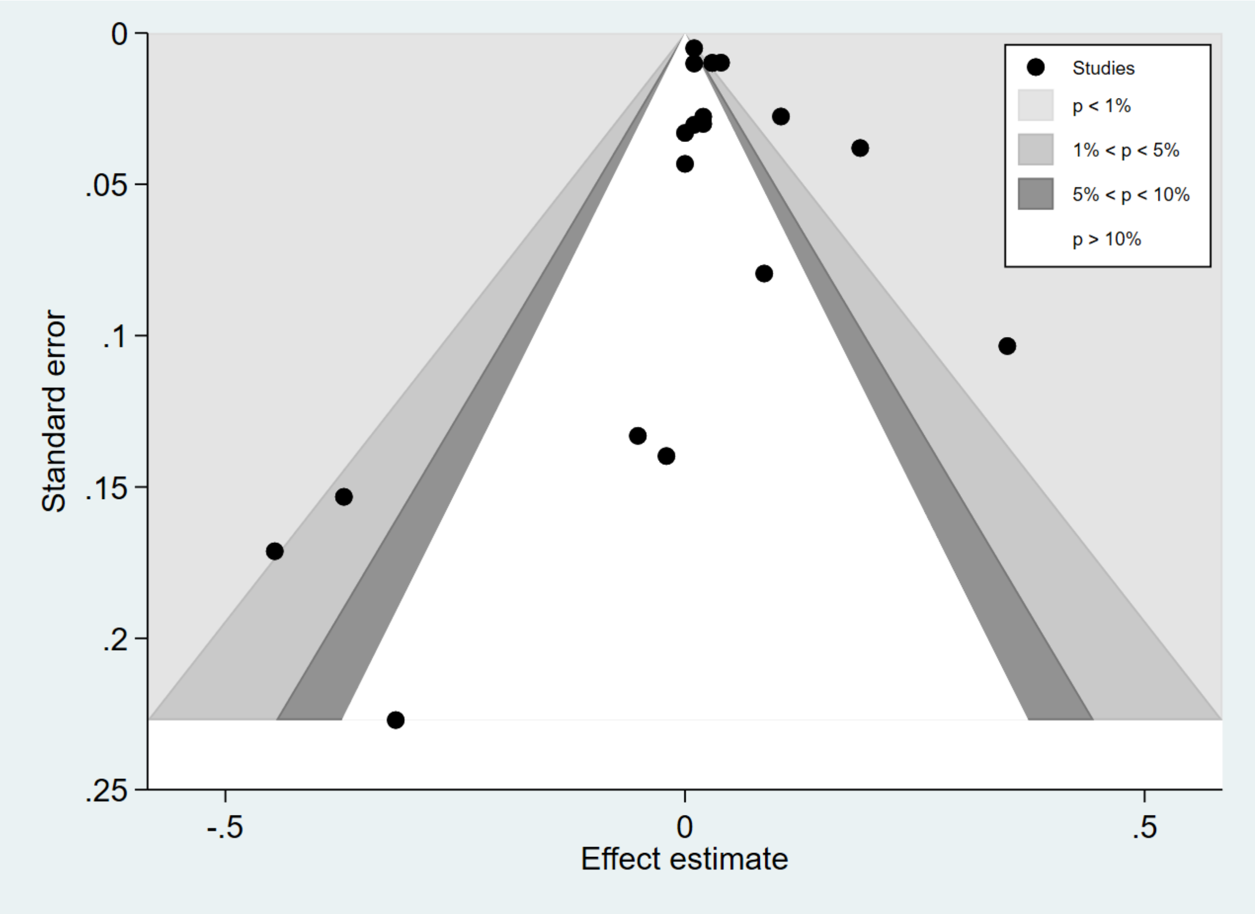

Appendix Figure S2. Cumulative Meta-analysis for Short-Term Mortality

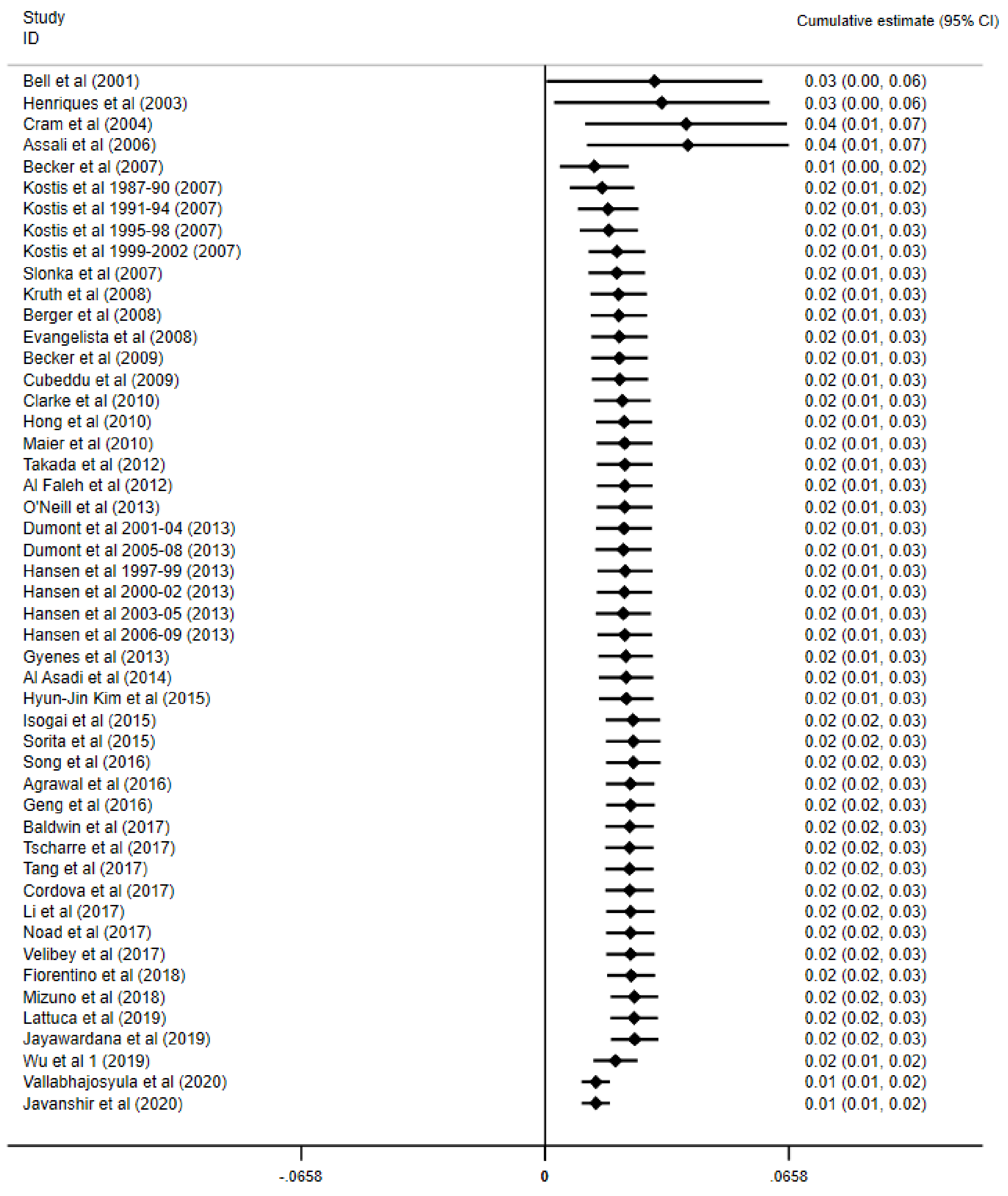

Appendix Table S4. Sensitive Analysis for short-Term Mortality

Study Included	Number of studies	I^2	OR (95%CI)	*P* Value
Only unadjusted data	13	83.5%	1.09 (1.03-1.14)	0.002
Only adjusted data	13	69.6%	1.06 (1.03-1.09)	0.000

CI, interval confidence

Appendix Table S5. GRADE Evidence Profile

Certainty assessment							№ of patients		Effect		Certainty	Importance
№ of studies	Study design	Risk of bias	Inconsistency	Indirectness	Imprecision	Other considerations	out of hours	on hours	Relative (95% CI)	Absolute (95% CI)		
**In-hospital or 30-day mortality (assessed with: OR)**												
49	observational studies	very serious [a,b,c]	serious [d]	serious [e]	serious [f]	publication bias strongly suspected [g]	-	-	**OR 1.04** (1.02 to 1.05)	**1 fewer per 1,000** (from 1 fewer to 1 fewer)	⊕◯◯◯ VERY LOW	CRITICAL
**1-year mortality (follow up: 1 year; assessed with: OR)**												
17	observational studies	serious [h]	serious [i]	serious [j]	serious [k]	none	-	-	**OR 1.03** (1.01 to 1.04)	**1 fewer per 1,000** (from 1 fewer to 1 fewer)	⊕◯◯◯ VERY LOW	CRITICAL

CI: Confidence interval; **OR:** Odds ratio

Explanations

a. existing publication bias
b. having substantial heterogeneity.
c. existing the risk of ecological bias.
d. some of included studies showed inconsistent results.
e. The included individuals have different comorbidity, and they did not adjust at the same level.

f. Although we did the subgroup analysis, we still cannot find the the resource of heterogeneity.

g. We did the contour-enhanced funnel plot and examined by a modified Macsskill test, found existing publication bias.

h. Some of included studies were analysed different AMI type, so we pooled STEMI or NSTEMI together.

i. Some of included studies showed inconsistent results

j. The included individuals have different comorbidity, and they did not adjust at the same level.

k. Although we did the subgroup analysis, we still can not find the the resource of heterogeneity.
