# Supplementary material for: Association Between Out-of-Hour Admission and Short- and Long-Term Mortality in Acute Myocardial Infarction: A Systematic Review and Meta-Analysis
Source: Front Cardiovasc Med. 2021 Dec 14;8:752675. doi: 10.3389/fcvm.2021.752675 (PMC8712470; doi:10.3389/fcvm.2021.752675)
Supplement: Supplementary file 2 [file Table_1.DOCX]

| Author, Year | Corresponding author country | Study period | MI types | Reperfusion Therapy | Outcomes | Adjusted Confounders | NOS | Sample  Size |
| --- | --- | --- | --- | --- | --- | --- | --- | --- |
| Vallabhajosyula et al 2020 | US | 2000-2016 | STEMI  NSTEMI | PCI | In-hospital mortality | age, sex, race, comorbidity, primary payer, hospital  region, hospital location and teaching status, hospital bed count, cardiogenic shock, and cardiac arrest | 9 | 9,041,819 |
| Javanshir et al 2020 | Iran | 2012.03-2017.02 | STEMI | PPCI | In-hospital mortality | diabetes, hypertension | 8 | 300 |
| Lattuca et al  2019 | France | 2003.01-2013.09 | STEMI | PPCI | In-hospital and 1-year mortality | age, smoking status,  family history of coronary artery disease, blood pressure at admission, heart rate, previous MI, beta-blocker treatment, ACE inhibitor (separately) | 7 | 2167 |
| Jayawardana et al. 2019 | UK | 2007.01-2012.12 | STEMI | PPCI | 30-day and in-hospital mortality | AMG risk score, sex, Index of Multiple Deprivation score,  previous AMI, angina, peripheral vascular disease, stroke, PCI, CABG, CKD, diabetes, smoking status, hypercholesterolemia, hypertension, asthma/COPD,  family history of coronary heart disease, annual hospital PPCI volume and month and year of admission | 8 | 76,648 |
| Chien et al  2019 | China | 2000-2009 | not specified | TPA; PTCA; CABG | 1-year all-cause mortality | weekends, age, Charlson comorbidity index (CCI), thrombolytic therapy | 7 | 2,007 |
| Wu et al 2019 | UK | 2004.01.01-2013.03.31 | STEMI; NSTEMI | PCI, CABG, any revascularization | In-hospital mortality | case mix, comorbidities, clinical presentations, treatments, and medications. | 8 | 615,035 |
| Dharma et al  2018 | Indonesia | 2008-2013 | STEMI | PPCI | all-cause mortality at one and two years | Age, gender, location of MI, diabetes, hypertension, Killip Class, TIMI score | 8 | 1,126 |
| Fiorentino et al 2018 | Portugal | 2011-2015 | STEMI; NSTEMI | PCI, CABG, any revascularization | In-hospital mortality | Age, female, cardiac procedure, influenza, residence match, stroke, cardiac dysrhythmias, acute renal failure, shock | 7 | 44,820 |
| Mizuno et al 2018 | Japan | 2011.04-2015.03 | not specified | PCI, CABG | In-hospital all-cause mortality | gender, age-group, and Killip classification | 7 | 103,908 |
| Eindhoven et al 2018 | Netherlands | 2012-2013 | STEMI  NSTEMI | PCI, other revascularization | 1-year mortality | - | 7 | 59,534 |
| Baldwin et al  2017 | Australia | 2009.06-2012.06 | not specified | NR | 30-day in or out of hospital mortality | age, gender | 8 | 148, 722 |
| Tscharre et al  2017 | Austria | 2003-2009 | STEMI | PPCI | 30-day all-cause mortality | age, gender, diabetes, hypertension, hyperlipidaemia, status of smoking, previous MI, familiar history of coronary heart disease (CHD), cardiogenic shock, anterior wall infarction and treatment modality | 8 | 2,829 |
| Tang et al  2017 | China | 2010.01-2015.12 | STEMI | PPCI | in-hospital mortality and MACEs | Symptom onset to balloon time, DTB time, Killip class IV, Ventricular septal rupture, Post-PCI TIMI flow (0–1) (separately) | 7 | 441 |
| Cordova et al  2017 | US | 2008.01.01-2010.01.31 | not specified | NR | In-hospital mortality | Age, hospital technology status, nurse staffing | 7 | 1,343 |
| Li et al  2017 | China | 2011.12-2014.12 | STEMI | PPCI; Fibrinolysis | In-hospital all-cause mortality | Stroke, obesity, Disease severity include systolic blood pressure<90mmHg, heart rate, Killip class,  cardiopulmonary resuscitation and abnormal rhythm of heart within 24 hours of admission, thrombolytic therapy, aspirin and angiotensin converting enzyme inhibitors /  angiotensin receptor blocker within 24 hours of admission | 7 | 7,456 |
| Noad et al 2017 | UK | 2009.04-2015.09 | STEMI; NSTEMI | PCI, other revascularizations | 30-day and in-hospital mortality | type of infarction, age and  gender | 7 | 3,757 |
| Velibey et al 2017 | Turkey | 2009.01-2014.12 | STEMI | PPCI | In-hospital mortality | Age, female, creatinine, hypertension, LVEF<40%, diabetes mellitus (separately) | 7 | 2,552 |
| Song et al  2016 | China | 2012.04-2015.03 | STEMI | PPCI | In-hospital mortality | Age, gender, stroke, heart rate, beta-blocker treatment, (separately) | 7 | 289 |
| Agrawal et al  2016 | US | 2003-2011 | NSTEMI | PPCI | In-hospital mortality | - | 8 | 3,625,271 |
| Geng et al  2016 | China | 2012.01-2015.12 | STEMI | PPCI | In-hospital mortality | age, creatinine, systolic BP, heart rate, symptom-to-door time, diabetes mellitus, hemoglobin, glucose, TIMI flow 2–3 after PCI and multivessel disease | 8 | 1,594 |
| Hyun-Jin Kim et al 2015 | Korea | 2007.01-2010.12 | NSTEMI; UA | PCI | 30-day cardiac mortality | Age, male, hypertension, diabetes mellitus, hyperlipidemia, smoking, previous MI, previous CABG (separately) | 7 | 577 |
| Isogai et al 2015 | Japan | 2010.07.01-2013.03.31 | not specified | PCI, CABG, any revascularization | In-hospital and 30-day mortality | age, sex, ambulance use, Killip class,  nine comorbidities present at admission, type of hospital, hospital volume, reperfusion therapies on the day of admission, mechanical support on the day of admission, revascularization  procedures during hospitalization, and drugs during hospitalization | 8 | 111,200 |
| Sorita et al 2015 | US | 1998.01-2010.06 | STEMI; NSTEMI | PCI | In-hospital and 30-day mortality | age, cardiogenic shock before PCI, MI onset to PCI within 24 hours, serum creatinine, left ventricular ejection fraction, congestive  heart failure (CHF) on presentation without shock, and peripheral artery disease | 8 | 6,086 |
| Al‑Asadi et al  2014 | Iraq | 2012.01.01-2012.09.30 | STEMI; NSTEMI | PCI, CABG, any revascularization | In-hospital mortality | Age, female, time of admission, diabetes mellitus, complications, low diastolic BP on admission | 8 | 419 |
| O'Neill et al  2013 | Canada | 2005.04.01-2010.10.31 | NSTEMI | PPCI; CABG | In-hospital, 30-day and 1-year mortality | - | 8 | 11,981 |
| Dumont et al  2013 | France | 2001-2004; 2005-2008 | STEMI | PCI, CABG, any revascularization | 30-day and 1-year cardiovascular mortality | Emergency Medical Dispatch Centers implementation, LVEF, GRACE risk score | 7 | 3,376 |
| Hansen et al  2013 | Denmark | 1997.01.01-2009.12.31 | not specified | PCI and CABG | 30-day and 1-year all-cause mortality | age, sex and coexisting comorbidities (HF, cardiac dysrhythmia, pulmonary edema, cardiogenic shock, cerebrovascular disease, COPD, cancer) | 8 | 101,948 |
| Gyenes et al  2013 | Canada | 1999.06-2008.12 | NSTEMI | PCI, CABG, any revascularization | In-hospital mortality | age, Killip class, systolic BP, heart rate, initial creatinine, cardiac arrest at presentation, ST deviation, positive initial cardiac enzyme markers,  history of TIA/stroke and on-site coronary angiography | 8 | 6,711 |
| Takada et al  2012 | Brazil | 2004.01-2007.06 | STEMI; NSTEMI; UA | PCI, CABG, any revascularization | In-hospital mortality | Age, EF, surgical treatment (separately) | 7 | 1,104 |
| Al Faleh et al 2012 | Saudi Arabia | 2005.12-2007.12 | STEMI; NSTEMI; UA | PCI, CABG, any revascularization | In-hospital mortality | - | 7 | 1,139 |
| Clarke et al 2010 | Australia | 2002/2003–2006/2007 | STEMI; NSTEMI | PCI, CABG, any revascularization | 30-day in-hospital mortality | Age, gender, indigenous status, remote residence,  socioeconomic status, comorbidities | 8 | 17,910 |
| Hong et al 2010 | Republic of Korea | 2003-2007 | STEMI; NSTEMI | PCI, CABG, any revascularization | 30-day mortality | Age, gender, comorbidities, length of stay, complications | 8 | 97,466 |
| Maier et al  2010 | Germany | 2004-2007 | STEMI | PCI | In-hospital mortality | Age, female, cardiogenic shock at admission, diabetes mellitus, renal failure, previous MI, D2B time (separately) | 8 | 2,131 |
| Becker et al 2009 | Hungary | 2003.01.01-2005.12.31 | STEMI | PPCI | 30-day and 1-year mortality | Age, gender | 8 | 1,890 |
| Cubeddu et al 2009 | US | 2003-2007 | STEMI | PPCI | In-hospital mortality | Age, body surface area, gender, family history of coronary artery, disease, smoking, comorbidities, serum creatinine | 8 | 747 |
| Kruth et al  2008 | Germany | 1994-2002 | STEMI | PPCI; Fibrinolysis | In-hospital mortality | age, gender, previous MI, diabetes, hypertension, prehospital delay, tachycardia (heart rate > 100/min) | 9 | 11,516 |
| Berger et al  2008 | Switzerland | 1997.01.01-2006.03.30 | STEMI | Thrombolysis; PPCI | In-hospital mortality | Age, Killip II-IV | 9 | 12,480 |
| Evangelista et al 2008 | Brazil | 2002 | STEMI; NSTEMI | PCI | In-hospital and 1-year mortality | Age, sex, admission specialty,  intensive care unit admission,  hospital type, direct admission | 7 | 869 |
| Becker et al 2007 | US | 1989-1998 | not specified | PCI, CABG, any revascularization | 30-day and 1-year mortality | - | 7 | 922,074 |
| Kostis et al 2007 | US | 1987-2002 | not specified | PCI, CABG | 30-day ,1-year and in-hospital mortality | age, sex, site of myocardial infarction, and coexisting conditions (diabetes, hypertension, renal disease, liver disease, anemia, cancer, cerebrovascular disease) | 8 | 231,164 |
| Slonka et al 2007 | Poland | 1998.01-2003.10 | STEMI | PPCI | In-hospital mortality | - | 7 | 1,778 |
| Assali et al 2006 | Israel | 2001.01-2004.06 | STEMI | PPCI | In-hospital and 30-day mortality | Age, left ventricular ejection fraction, anemia, renal failure, Killip class, final TIMI flow,  number of coronary vessels diseases | 8 | 273 |
| Cram et al 2004 | US | 1998 | STEMI; NSTEMI | NR | In-hospital mortality | Age, gender, race, comorbidities | 7 | 42,974 |
| Henriques et al 2003 | Netherlands | 1994-2000 | STEMI | PPCI | 30-day mortality | - | 7 | 1,702 |
| Bell et al  2001 | Canada | 1988.04.01-1997.03.31 | not specified | NR | In-hospital mortality | age, gender | 8 | 3,789,917 |

MI, myocardial infarction; STEMI, ST-elevation myocardial infarction; PCI, percutaneous coronary intervention; CABG, coronary artery bypass graft; DTB: door-to-balloon; TIMI: thrombolysis in myocardial infarction.
